# Immunothrombosis and new-onset atrial fibrillation in the general population: the Rotterdam Study

**DOI:** 10.1007/s00392-021-01938-4

**Published:** 2021-09-24

**Authors:** Martijn J. Tilly, Sven Geurts, Samantha J. Donkel, M. Arfan Ikram, Natasja M. S. de Groot, Moniek P. M. de Maat, Maryam Kavousi

**Affiliations:** 1 Department of Epidemiology, Erasmus MC University Medical Center Rotterdam, Rotterdam, The Netherlands; 2 Department of Hematology, Erasmus MC University Medical Center Rotterdam, Rotterdam, The Netherlands; 3 Department of Cardiology, Erasmus MC University Medical Center Rotterdam, Office Na-2714, PO Box 2040, 3000 CA Rotterdam, The Netherlands

**Keywords:** Immunothrombosis, Atrial fibrillation, NETs, Fibrinogen, Von Willebrand factor, ADAMTS13

## Abstract

**Background:**

Atrial fibrillation (AF) is the most common age-related cardiac arrhythmia. The etiology underlying AF is still largely unknown. At the intersection of the innate immune system and hemostasis, immunothrombosis may be a possible cause of atrial remodeling, and therefore be an underlying cause of AF.

**Methods:**

From 1990 to 2014, we followed participants aged 55 and over, free from AF at inclusion. Immunothrombosis factors fibrinogen, von Willebrand factor, ADAMTS13, and neutrophil extracellular traps (NETs) levels were measured at baseline. Participants were followed until either onset of AF, loss-to-follow-up, or reaching the end-date of 01-01-2014. Cox proportional hazard modelling was used to calculate hazard ratios (HRs) and 95% confidence intervals (CIs), adjusted for cardiovascular risk factors.

**Results:**

We followed 6174 participants (mean age 69.1 years, 57% women) for a median follow-up time of 12.8 years. 364 men (13.7%, incidence rate 13.0/1000 person-years) and 365 women (10.4%, incidence rate 8.9/1000 person-years) developed AF. We found no significant association between markers of immunothrombosis and new-onset AF after adjusting for cardiovascular risk factors [HR 1.00 (95% CI 0.93–1.08) for fibrinogen, 1.04 (0.97–1.12) for von Willebrand factor, 1.00 (1.00–1.01) for ADAMTS13, and 1.01 (0.94–1.09) for NETs]. In addition, we found no differences in associations between men and women.

**Conclusion:**

We found no associations between markers of immunothrombosis and new-onset AF in the general population. Inflammation and immunothrombosis may be associated with AF through other cardiovascular risk factors or predisposing conditions of AF. Our findings challenge the added value of biomarkers in AF risk prediction.

**Graphic abstract:**

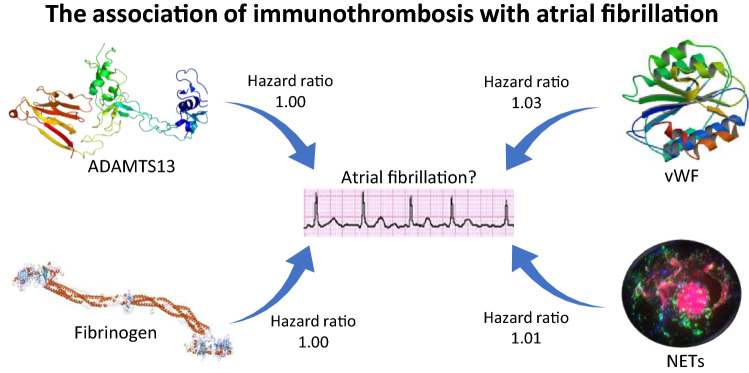

**Supplementary Information:**

The online version contains supplementary material available at 10.1007/s00392-021-01938-4.

## Introduction

Atrial fibrillation (AF) is the most common cardiac arrhythmia of clinical significance [1]. Despite the high prevalence, the etiology underlying AF is still largely unknown. Atrial remodeling is among the pathways promoting initiation and perpetuation of AF [2]. It is hypothesized that inflammation is one of the underlying conditions of atrial remodeling and AF [3, 4].

Immunothrombosis refers to the complex participation of the innate immune system in the formation of intravascular thrombus through distinct cellular and molecular interactions [5–7]. This local coagulation can promote more inflammatory processes, initiating atrial remodeling through direct and indirect tissue damage [3, 4]. Fibrinogen, von Willebrand factor (vWF), and A Disintegrin and Metalloprotease with ThromboSpondin motif repeats 13 (ADAMTS13), a vWF-cleaving protease, are biomarkers that play key roles in coagulation and inflammatory pathways, and may therefore be associated with AF [8–10]. However, prospective research on this is scarce.

Activation of the innate immunity can cause neutrophils to release neutrophil extracellular traps (NETs) [5, 6, 11]. Besides their important role in actively killing pathogens by releasing chromatin and DNA [12], NETs also stimulate coagulation processes by recruiting and activating platelets, binding to tissue factor, and stimulating fibrinogen and vWF [5, 7, 13, 14]. This way, NETs are at the intersection between inflammation and thrombosis, both potentially major players in AF pathophysiology. However, the association of NETs and new-onset AF has not been investigated.

We aim to investigate the association between markers of immunothrombosis, including fibrinogen, vWF antigen (vWF:Ag), ADAMTS13, vWF:Ag/ADAMTS13 ratio, and NETs, with the risk of new-onset AF among community-dwelling men and women from the large population-based Rotterdam Study.

## Materials and methods

### Study population

A description of the study population is available in Online Resource 1. Briefly, this study consists of men and women participating in the Rotterdam Study, an ongoing large, prospective population-based cohort study among inhabitants of Ommoord, a suburb in Rotterdam, the Netherlands [15]. We included 6174 participants free of AF at baseline who underwent blood sampling tests for fibrinogen, vWF:Ag, ADAMTS13, or MPO–DNA complex levels (Online Resource 2).

### Assessment of markers of immunothrombosis

Fibrinogen levels were derived from the clotting curve of the prothrombin time assay, using Thromborel S (Behringwerke, Marburg, Germany) on the ACL 300 coagulation analyzer (Instrumentation Laboratory). vWF:Ag levels were measured with an in-house ELISA using polyclonal rabbit antihuman VWF antibodies and horseradish-peroxidase-conjugated antihuman VWF antibodies (DakoCytomation, Glostrup, Denmark) to catch and tag vWF. ADAMTS13 activity was measured in a kinetic assay using Fluorescence Resonance Energy Transfer Substrate VWF 73 (FRETS-VWF73), as is thoroughly described in the previous articles [16, 17].

We determined NET levels by measuring MPO–DNA complexes with an ELISA as reported earlier [18]. We adjusted the commercial human cell death ELISA kit (Cell death detection ELISAPLUS, Roche Diagnostics Nederland B.V., Almere, The Netherlands). Briefly, as the capturing antibody, we used anti-MPO monoclonal antibody (clone 4A4, ABD Serotec). Patient plasma was added in combination with the peroxidase-labeled anti-DNA monoclonal antibody (from cell death detection ELISA kit; Roche). The absorbance at 405 nm wavelength was measured using Biotek Synergy HT plate reader with a reference filter of 490 nm. The values are expressed as milli-arbitrary units (mAU/mL).

### Assessment of atrial fibrillation

AF was defined in accordance with the European Society of Cardiology (ESC) guidelines [1]. At study entry, prevalent AF and other diseases are assessed by an extensive interview and review of medical records. During the follow-up, participants are continuously monitored through a linkage of the study database with medical records of general practitioners and hospitals. The date of incident AF was defined as the date of the first occurrence of symptoms suggestive of AF with subsequent electrocardiogram (ECG) verification. At baseline and follow-up examinations, ten second 12-lead ECGs were taken and stored digitally with an ACTA Gnosis IV ECG recorder (Esaote; Biomedical, Florence Italy). All ECGs were analyzed using Modular ECG Analysis System (MEANS), a software system that has been described previously [19]. The ECGs diagnosed by MEANS as rhythm disorder were independently verified by two research physicians blind to the MEANS diagnosis. A cardiologist was consulted in case of disagreement. Events of AF were not included if these occurred during the process of dying, or in case of transient AF after cardiac surgery or myocardial infarction (MI). Participants were followed from the inclusion date until date of onset of AF, loss to follow-up, date of death, or January 1st 2014, whichever occurred first.

### Assessment of cardiovascular risk factors

We collected the data on body mass index (BMI), smoking, alcohol use, renal function, differential blood count, hypertension, cardiac therapy, lipid-reducing agents, prevalent coronary heart disease (CHD), heart failure (HF), and diabetes mellitus (DM). A complete description of the assessment of cardiovascular risk factors is available in Online Resource 1.

### Statistical analysis

Baseline characteristics were presented as counts and percentages or mean and standard deviation (SD), or median and inter-quartile range (IQR) in case of skewedness. Incidence rates are presented as events per 1000 person-years (py). Differences between men and women were assessed through Independent Samples T tests, Mann–Whitney *U* tests, and Pearson’s Chi-square tests. Because of skewed distributions, values for fibrinogen, vWF:Ag and MPO–DNA complexes were transformed using the natural logarithm (Ln). Each marker was standardized to obtain hazard ratios (HRs) and 95% confidence intervals (CIs) per 1-SD increment. We determined the quartiles of fibrinogen, vWF:Ag, ADAMTS13, and MPO–DNA complexes. For fibrinogen, vWF:Ag, and MPO–DNA complexes the first quartiles were used as reference quartile. For ADAMTS13, the fourth quartile was used as reference. To examine the combination of vWF:Ag levels and ADAMTS13 activity on AF incidence, we combined vWF:Ag levels above or below the 75th percentile, and ADAMTS13 activity levels above and below the 25th percentile.

Univariable and multivariable Cox proportional hazards regression analyses were performed. Models were adjusted for age, sex, and cohort (model 1), and additionally for cardiovascular risk factors including: current smoking, alcohol use, estimated glomerular filtration rate (eGFR), hypertension, use of cardiac therapy, use of lipid-reducing agents, prevalent DM, prevalent HF, and prevalent CHD (model 2). HRs and 95% CIs were calculated to quantify the associations. The proportional hazard assumptions were tested by Schoenfeld residual testing and found to be satisfied. Missing values of covariates (range 0.0–4.9%) were imputed under the assumption of missing at random. All available data were used to generate five imputed datasets. The results from each imputed dataset were combined to present single estimates. In addition, analyses were performed in men and women separately.

Statistical significance was considered at two-tailed *p* value ≤ 0.05. All analyses and data management were done with IBM SPSS Statistics for Windows, version 25.0 (IBM Corp., Armonk, New York, USA) and R: A language and environment for statistical computing, version 4.0.3 (R Foundation for Statistical Computing, Vienna, Austria).

## Results

### Baseline characteristics

Baseline characteristics are presented in Table 1. We included 6174 participants (mean age 69.1 ± 8.2 years), of whom 3520 (57.0%) were women. Median blood levels for fibrinogen, vWF:Ag, and MPO**–**DNA complexes were 3.8 g/L, 1.19 IU/mL, and 53 mAU/mL, respectively. The mean plasma level for ADAMTS13 activity was 91.6% ± 17.7%.

**Table 1:** Baseline characteristics of the study population

	Total population (*N* = 6174)
Age, years	69.1 ± 8.2
BMI (kg/m^2^)	27.0 ± 4.3
Current smoker, *N*(%)	1247 (20.2%)
Prevalent DM, *N*(%)	797 (12.9%)
Prevalent CHD, *N*(%)	518 (8.4%)
Prevalent HF, *N*(%)	163 (2.6%)
Prevalent hypertension, *N*(%)	4163 (67.4%)
eGFR (mL/min/1.73 m^2^)	74.9 ± 15.7
Systolic blood pressure (mmHg)	143.3 ± 21.2
Diastolic blood pressure (mmHg)	76.8 ± 11.1
Blood pressure lowering medication, *N*(%)	2172 (35.2%)
Daily alcohol intake (g)	5.0 (17.0)
Prevalent alcohol abuse, *N*(%)	939 (15.2%)
Use of cardiac therapy, *N*(%)	484 (7.8%)
Lipid-reducing agents, *N*(%)	812 (13.2%)
Thrombocyte count (× 10^9^/L)	257.4 ± 58.3
Leucocyte count (× 10^9^/L)	6.8 ± 1.9
Lymphocyte count (× 10^9^/L)	2.6 ± 1.0
Lymphocyte percentage of leucocytes (%)	38.6 ± 7.8
Platelet to lymphocyte ratio	106.3 ± 34.5
Total cholesterol (mmol/L)	5.8 ± 1.0
HDL cholesterol (mmol/L)	1.4 ± 0.4
CRP (mg/L)	1.7 (3.0)
Plasma fibrinogen (g/L)	3.8 (1.1)
Plasma VWF:Ag (IU/mL)	1.19 (0.66)
ADAMTS13 activity (%)	91.6 ± 17.7
MPO–DNA complex (mAU/mL)	53 (45)

Categorical data presented as *N* (%)

Continuous data presented as mean ± SD for normally distributed data, or median (IQR) for skewed distributed data

*BMI* Body Mass Index, *DM* diabetes mellitus, *CHD* coronary heart disease, *HF* heart failure, *eGFR* estimated glomerular filtration rate, *HDL* high density lipoprotein, *CRP* C-reactive protein, *vWF:Ag* von Willebrand Factor antigen, *ADAMTS13* A Disintegrin And Metalloprotease with ThromboSpondin motif repeats 13. Alcohol abuse is defined as ≥ 4 alcoholic consumptions/day for men, and ≥ 2 for women

Women were significantly older (mean age 69.6 ± 8.4 years vs 68.4 ± 7.7 years) and had a higher mean BMI (27.3 ± 4.4 vs 26.5 ± 3.7 kg/m^2^), whereas men had significantly higher prevalence of DM (14.9% vs 11.4%) and CHD (14.2% vs 4.0%). Differential bloodwork showed significant differences for thrombocyte count (241.4 ± 55.7 vs 269.4 ± 57.4 × 10^9^/L), leucocyte count (7.0 ± 1.9 vs 5.7 ± 1.9 × 10^9^/L), lymphocyte count (2.6 ± 0.9 vs 2.6 ± 1.0 × 10^9^/L), lymphocyte percentage of total leucocytes (37.5 ± 7.5 vs 39.4 ± 8.0%), and platelet to lymphocyte ratio (99.1 ± 32.3 vs 111.6 ± 35.1) between men and women, respectively. Median fibrinogen (3.9 g/L [IQR 1.1] for men versus 3.7 g/L [IQR 1.1] for women) and ADAMTS13 activity (94.8% ± 17.6% for men vs 87.3% ± 16.9% for women) were significantly different between men and women (Online Resource 3).

### Incident atrial fibrillation

During a median follow-up of 12.8 (IQR 5.6) years (69093py), 729 participants (364 men and 365 women) developed AF (incident rate 10.6 /1000 py). Incidence rates were 13.0/1000py for men and 8.9/1000py for women. There were no significant associations between fibrinogen [HR (95% CI): 1.00 (0.93–1.08)], vWF:Ag [HR (95% CI): 1.03 (0.95–1.11)], ADAMTS13 [HR (95% CI): 1.00 (1.00–1.01)], vWF:Ag/ADAMTS13 ratio [HR (95% CI)): 1.00 (0.93–1.08)], or MPO–DNA complexes [HR (95% CI): 1.01 (0.94–1.09)] with new-onset AF after adjustments (Table 2).

**Table 2:** Association between markers of immunothrombosis and incident atrial fibrillation in the total population

	Model 1 HR (95% CI)	Model 2 HR (95% CI)
Fibrinogen (g/L)	1.02 (0.95–1.10)	1.00 (0.93–1.08)
vWF:Ag (IU/mL)	1.05 (0.97–1.13)	1.03 (0.95–1.11)
ADAMTS13 (%)	1.00 (1.00–1.01)	1.00 (1.00–1.01)
vWF:Ag/ADAMTS13 ratio	1.02 (0.95–1.10)	1.01 (0.93–1.08)
MPO–DNA complex (mAU/mL)	1.01 (0.94–1.09)	1.01 (0.94–1.09)

Presented estimated are hazard ratio (95% confidence interval) per one standard deviation increase of each immunothrombosis marker

Model 1 is adjusted for age, sex, and Rotterdam Study cohort

Model 2 is additionally adjusted for current smoking, alcohol use, renal function, hypertension, use of cardiac therapy, use of lipid-reducing agents, prevalent diabetes mellitus, prevalent heart failure, and prevalent coronary heart disease

*HR* Hazard Ratio, *CI* Confidence Interval, *vWF:Ag* von Willebrand Factor antigen, *ADAMTS13* A Disintegrin And Metalloprotease with ThromboSpondin motif repeats 13

Univariable Cox proportional hazard regression showed a significant larger risk of new-onset AF with higher levels of vWF:Ag in both men [HR (95% CI): 1.19 (1.07–1.32)] and women [HR (95% CI): 1.14 (1.03–1.27)]. After adjusting for cardiovascular risk factors, the associations attenuated (Online Resource 4). For fibrinogen, ADAMTS13, and MPODNA complexes we found no associations in men or women.

**Fig. 1:**
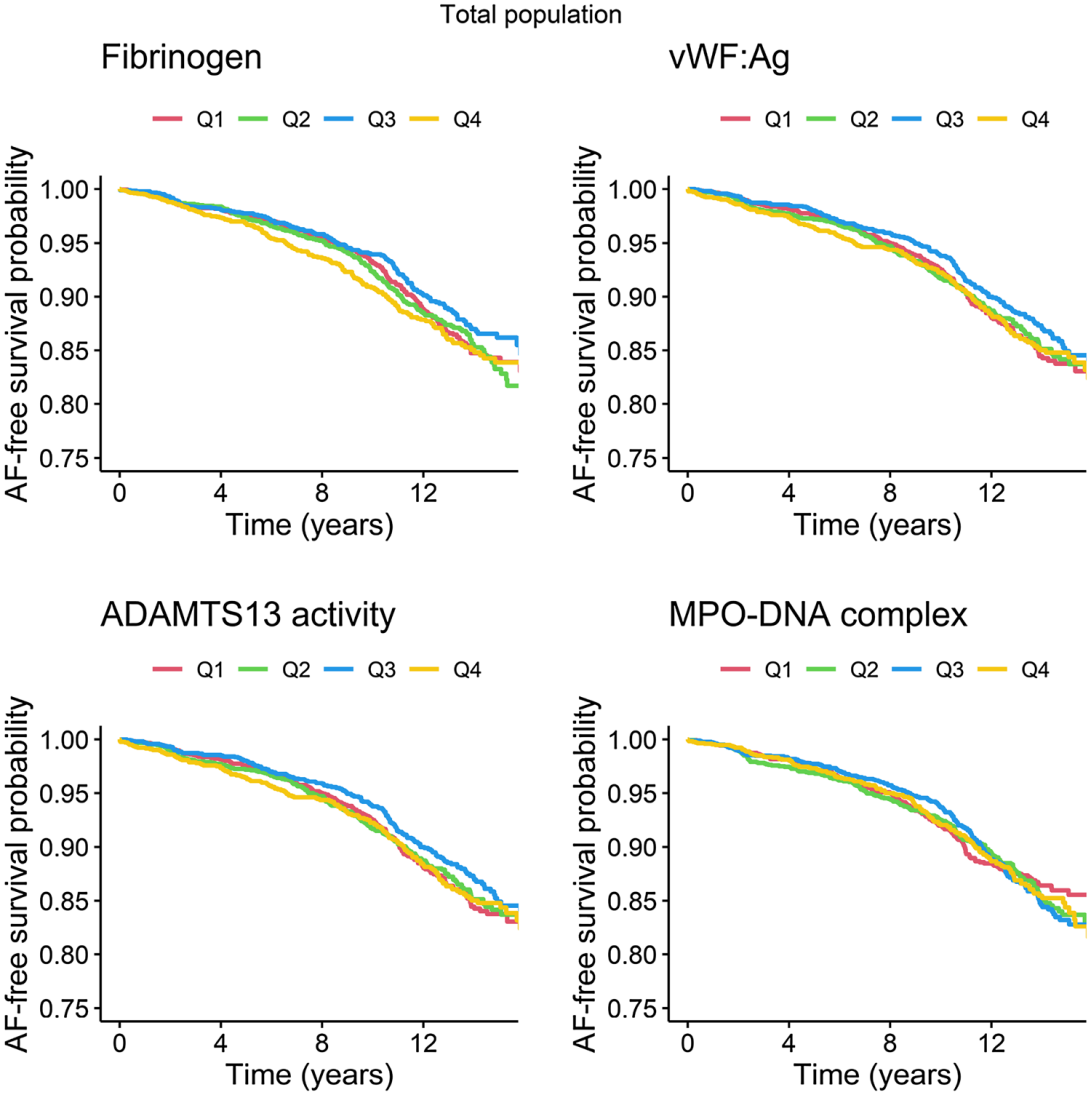
Association between markers of immunothrombosis and incident atrial fibrillation in the total population, per quartile. Adjusted for age, sex, Rotterdam Study cohort, current smoking, alcohol use, renal function, hypertension, use of cardiac therapy, use of lipid-reducing agents, prevalent diabetes mellitus, prevalent heart failure, and prevalent coronary heart disease. Quartiles fibrinogen: ≤ 3.30 g/L, 3.31–3.80 g/L, 3.81–4.40 g/L, and ≥ 4.41 g/L. Quartiles vWF:Ag: ≤ 0.93 IU/mL, 0.94–1.20 IU/mL, 1.21–1.60 IU/mL, and ≥ 1.61 IU/mL. Quartiles ADAMTS13: ≤ 80.31%, 80.32–91.00%, 91.01–101.75%, and ≥ 101.76%. Quartiles MPO–DNA complex:  ≤ 42 mAU/mL,42–53 mAU/mL, 54–87 mAU/mL, and 88 mAU/mL

Both univariable analysis and multivariable analysis showed no significant differences in risk between quartiles for fibrinogen or MPO–DNA complexes (Fig. 1). We found a higher risk of new-onset AF with vWF:Ag levels in the highest quartile as compared to the lowest quartile [HR (95% CI): 1.37 (1.11–1.70)], and for ADAMTS13 levels in the lowest quartile, as compared to the highest [HR (95% CI): 1.51 (1.23–1.86)] in univariable models. After adjustment for cardiovascular risk factors, the associations attenuated (Fig. 1).

In sex-stratified analyses, highest versus lowest vWF:Ag levels showed a significant larger AF risk in women [HR (95% CI): 1.55 (1.16–2.07)] in univariable analysis, but not in men. In contrast, the lowest versus highest ADAMTS13 activity levels were associated with AF risk among men [HR (95% CI): 1.63 (1.20–2.22)] in univariable analysis, but not in women. After adjustment for cardiovascular risk factors, the associations were not statistically significant (Online Resource 5).

Combining vWF:Ag and ADAMTS13 levels, participants with vWF:Ag levels ≥ 1.61 IU/mL and ADAMTS13 activity ≤ 80.31% had a significantly higher risk of new-onset AF than participants with vWF:Ag < 1.61 IU/mL and ADAMTS13 activity > 80.31% [HR (95% CI): 1.47 (1.09–1.98)], albeit nonsignificant after adjustments (Table 2). Sex-stratified analyses showed a larger risk for AF with vWF:Ag levels < 1.61 IU/mL and ADAMTS13 activity ≤ 80.31% [HR (95% CI): 1.55 (1.22–1.97)] in men in univariable analysis, but not in women. After adjustments, all associations attenuated (Online Resource 5).

## Discussion

In this prospective population-based study, we examined the association between immunothrombosis and new-onset AF among men and women. Biomarkers related to inflammation and coagulation, including fibrinogen, vWF, ADAMTS13, vWF:Ag/ADAMTS13 ratio, and NETs were not independently associated with new-onset AF.

This is the first large prospective population-based cohort study to evaluate the link between NET formation and new-onset AF development. Previous studies suggested that markers of systemic and local inflammation are associated with AF development [20, 21]. While the exact pathways of the development of AF are still unknown, immunothrombosis is implicated in AF pathophysiology. NETs play an important role in immunothrombosis. During the process of NETosis, histones, antimicrobial proteins, and cell-free DNA are released from cells, especially neutrophils [5, 6, 11, 12, 14]. Through Toll-like receptors (TLR) 2, 4, and 9, these histones cause inflammation and eventually cell-death in endothelial and epithelial cells [22]. The histones, as well as the DNA, also directly activate local platelets, which in turn activate the coagulation cascade [22]. Through these processes NETs can cause tissue injury by directly killing endothelial cells and through local microvascular thrombosis. The local tissue damage caused by NETs, combined with the inflammatory effects related to immunothrombosis, can lead to structural and electrical remodeling of the atria [2, 3, 23–28]. This progressively impairs atrial conduction and promote reentry, giving rise to AF [29, 30]. However, the lack of significant associations in our study suggests that the potential impact of inflammation on AF development lies in other pathways than the above-described paths of immunothrombosis. Therefore, more research on the role of immunothrombosis in the development of AF is required.

Fibrinogen, vWF, and ADAMTS13 play important roles in both coagulation and inflammation, and have been reported as independent risk factors for cardiovascular disease [9, 31–34]. Higher levels of fibrinogen and vWF can lead to intravascular thrombosis, vascular damage, and thrombotic complications, whereas lower levels of ADAMTS13 result in decreased cleavage of large prothrombotic vWF multimers [32]. Nonetheless, we did not find any association between these risk markers with incident AF among women and men from the general population.

Similar to our findings, fibrinogen was not associated with incidence of AF within the Framingham Offspring Study [35] and the Malmö Preventive Study [36]. However, fibrinogen showed significant associations with incident AF in the Copenhagen City Heart Study, the ARIC study, and the Women’s Health Study [37–39]. Higher levels of fibrinogen may indicate underlying inflammatory processes. Local inflammation may cause local remodeling of the atria, eventually disrupting the conduction, and be a pathophysiological cause of new-onset AF [40]. However, the results regarding the association of fibrinogen with new-onset AF remain inconclusive. A possible explanation for these discrepancies could be the differences in study populations. The Copenhagen City Heart Study was based on hospitalized AF patients, and therefore possibly represent the most symptomatic and severe AF cases [38]. The ARIC study [37] and the Women’s Healthy Study [39] studied younger cohorts. The latter also lacked periodical ECG screening for AF, thus participants with asymptomatic AF or less severe cases of AF may have been missed.

To our knowledge, we are also the first large prospective cohort study reporting on the combination of vWF and ADAMTS13 levels. The ARIC study [37] and the Framingham Offspring Study [41] have reported significant associations between vWF:Ag and AF. vWF is secreted by damaged endothelial cells and plays an active role in thrombogenesis by platelet aggregation [42, 43]. Thrombogenesis can cause further inflammation, cardiovascular complications, and oxidative stress, which can all be underlying causes of AF [44–46]. As ADAMTS13 degrades large, thrombogenic vWF-multimers into smaller and less thrombogenic molecules, an inverse association with AF is expected. A combination of higher levels for vWF:Ag and lower levels of ADAMTS13 may indicate underlying immunothrombosis. None of the previously mentioned studies looked at vWF:Ag and ADAMTS13 combined. The younger age of the participants in the ARIC study may partly explain differences between our results from the ones by ARIC investigators. Ko et al. used proteomics profiling to measure ADAMTS13 levels [41], whereas we measured ADAMTS13 activity using the functional FRETS assay [16, 17]. These different methods might, at least partly, explain different results.

After adjusting for additional cardiovascular risk factors, the observed associations between markers of immunothrombosis and AF attenuated. Possibly, inflammation and immunothrombosis are associated with AF through other cardiovascular risk factors or predisposing conditions to AF, such as CHD or HF. As factors of inflammation and hemostasis were previously associated with cardiovascular disease [31–34], the influence they have on AF initiation might be through these comorbidities. This way, the relation of inflammation and immunothrombosis with new-onset AF might be, partly, through other pathophysiological pathways than the direct effect of (local) inflammatory processes and atrial remodeling. In addition, immunothrombosis is a complex conjunction of the immune system and coagulation. Although we aimed to look at different aspects of immunothrombosis in this study, a connection between immunothrombosis and AF might be found through other pathways.

Recent studies have previously challenged the specificity and added value of various biomarkers in prediction of new-onset AF and AF complications [47, 48]. Most studies investigating the association of inflammatory biomarkers use specific patient populations. Therefore, elevated biomarkers may be representing the clinical situation or comorbidities of a patient, rather than actually having a causal relation with the investigated conditions. However, in a large, general population setting as in our current study, single biomarkers may not be specific enough to be of added value for AF prediction. Moreover, we carefully adjusted our analyses for relevant comorbidities and potential causes of confounding. This supports the notion that many biomarkers could often be a representation of the patient condition. While not investigated in this study, lack of specificity of biomarkers may also hold for the association of immunothrombotic biomarkers related to AF complications [48].

Recent evidence suggests sex differences in AF pathophysiology [49, 50]. While the incidence of AF is lower in women, women with AF have an increased risk of developing cardiovascular complication and mortality [49]. Sex differences in atrial remodeling and electrophysiological function have been reported [50]. It is known that autoimmune diseases are more prevalent in women, and immunologic differences between men and women have been reported [51, 52]. Also, the role of inflammation in AF initiation may be different in men and women. However, in our study we found no evidence of sex differences in the associations of immunothrombosis with AF.

The large population-based study population and long follow-up are the main strengths of this study. Through extensive interviews by trained interviewers, periodical research center visits, linkage with GP records, and meticulous adjudication of the events by study physicians, AF events are carefully assessed and a range of risk factors are available. However, there are also limitations. Despite the meticulous assessment we are unable to distinguish between paroxysmal and long-lasting AF as Holter monitoring is not available. Additionally, as blood was sampled at baseline, no inferences regarding longitudinal changes in markers and the effect of these changes on AF risk could be made. As our study shows, the development of AF greatly relies on other cardiovascular risks and patient conditions. As biomarkers, as well as many other cardiovascular risk factors, are dynamic, we can expect the evolution of these risk factors as individuals grow older will differ. Future studies investigating these biomarkers, taking into account their dynamic nature through repeated measurements and regular reassessments, are warranted to increase our knowledge regarding the link between AF and immunothrombosis. While representative of the general Dutch population above 55 years old, these results may not apply to men and women of younger age or other ethnicities. Lastly, we determined NET levels by measuring MPO–DNA complexes through ELISA. The specificity of ELISA to accurately detect NETs is controversial, and these results should therefore be cautiously interpreted [53].

## Conclusion

Fibrinogen, vWF:Ag, ADAMTS13, vWF:Ag/ADAMTS13, or NETs were not associated with the risk of new-onset AF in our large prospective population-based study. Our findings challenge the added value of biomarkers in AF prediction in a general population. Inflammation and immunothrombosis may be associated with AF through cardiovascular risk factors or other predisposing conditions to AF. Moreover, the impact of inflammation on new-onset AF could lie in other pathways than the examined immunothrombosis markers. Therefore, more prospective research towards markers of immunothrombosis in AF pathophysiology is warranted.

## Supplementary Information

Below is the link to the electronic supplementary material.

Supplementary file1 (PDF 474 kb)

Supplementary file2 (PDF 382 kb)

Supplementary file3 (PDF 396 kb)

Supplementary file4 (PDF 464 kb)

Supplementary file5 (PDF 705 kb)

## Data Availability

Data can be obtained upon request. Requests should be directed towards the management team of the Rotterdam Study (secretariat.epi@erasmusmc.nl), which has a protocol for approving data requests. Because of restrictions based on privacy regulations and informed consent of the participants, data cannot be made freely available in a public repository.
