# Supplementary material for: Immunothrombosis and new-onset atrial fibrillation in the general population: the Rotterdam Study
Source: Clin Res Cardiol. 2021 Sep 24;111(1):96–104. doi: 10.1007/s00392-021-01938-4 (PMC8766396; doi:10.1007/s00392-021-01938-4)
Supplement: Supplementary file 1 — Supplementary file1 (PDF 474 kb) [file 392_2021_1938_MOESM1_ESM.pdf]

Immunothrombosis and new-onset atrial fibrillation in the general population: the Rotterdam Study

Martijn J. Tilly[a], MD, Sven Geurts[a], MD, Samantha J. Donkel[b], MD, M. Arfan Ikram[a], Phd, Natasja M.S. de Groot[c], PhD, Moniek P.M. de Maat[b], PhD, *Maryam Kavousi[a], PhD

[a] Department of Epidemiology, Erasmus MC University Medical Center Rotterdam, Rotterdam, The Netherlands

[b] Department of Hematology, Erasmus MC University Medical Center Rotterdam, Rotterdam, The Netherlands

[c] Department of Cardiology, Erasmus MC University Medical Center Rotterdam, Rotterdam, The Netherlands

Corresponding author: Maryam Kavousi

Erasmus MC, University Medical Center Rotterdam, office Na-2714

PO Box 2040, 3000 CA Rotterdam, The Netherlands

Telephone Number: +31 10 7043997

Email: m.kavousi@erasmusmc.nl

Journal: Clinical Research in Cardiology

Online Resource 1.

Study population
The study population consisted of participants of the Rotterdam Study (RS), an ongoing large, prospective population-based cohort study among adult inhabitants of Ommoord, a suburb in Rotterdam, the Netherlands.[1] The aim of the RS is to obtain an overview of chronic diseases in mid-life and late-life in regards to risk factors, prognosis, etiology and potential intervention targets, by collecting data on determinants and occurrence of cardiovascular, neurological, ophthalmologic, locomotor, and psychiatric diseases. In 1990 the first cohort (RS-I) was enrolled in the study, consisting of 7,983 out of 10,215 eligible individuals. Baseline data was collected between 1990 and 1993 (RS-I-1), with follow-up examinations and questionnaires every 3 to 5 years. A new cohort (RS-II) was included in 2000, consisting of 3,011 participants out of the 4,472 inhabitants who moved to Ommoord or turned 55 since the start of RS-I. In 2006, another cohort was initiated (RS-III), including 3,932 out of 6,057 inhabitants aged 45-54 years. By the end of 2008, the study contained 14,926 participants aged 45 or older, out of 20,744 total inhabitants of Ommoord (overall response 72%) (Online Resource 2). Data on morbidity and mortality were continuously collected through linkage with digital files from general practitioners in the study area [1].

For this current study, we included participants from the third examination round of the first cohort (RS-I-3) and the first examination round of the second cohort (RS-II-1). We excluded all individuals without informed consent for follow-up data collection (n=251) or with current or a history of AF (n=632).
Out of 10,112 participants free of AF at baseline, 6,174 participants underwent blood sampling tests for fibrinogen, vWF antigen (vWF:Ag), ADAMTS13, or MPO-DNA complex blood levels and were included in this study.
The Rotterdam Study has been approved by the Medical Ethics Committee of the Erasmus MC (registration number MEC 02.1015) and by the Dutch Ministry of Health, Welfare, and Sport (Population Screening Act WBO, license number 1071272-159521-PG). The Rotterdam Study has been entered into the Netherlands National Trial Register (NTR; www.trialregister.nl) and into the WHO International Clinical Trials Registry Platform (ICTRP; www.who.int/ictrp/network/primary/en/) under shared catalog number NTR6831. All participants provided written informed consent to participate in the study and to have their information obtained from their treating physicians.

Assessment of cardiovascular risk factors
Extensive baseline data were obtained from participants at inclusion of the study through physical examinations and questionnaires. Trained research assistants conducted interviews for relevant information (e.g. medication use, smoking status). Body mass index (BMI) was defined as the weight in kilograms, divided by the square of length in meters. Blood pressure was defined as the mean systolic and diastolic pressure of the right brachial artery after two consecutive measurements in sitting position. We defined hypertension as a systolic blood pressure of 140mmHg or higher, or a diastolic blood pressure of 90mmHg or higher, or the use of blood pressure lowering medication. Alcohol use was measured in grams/day. Alcohol abuse was defined as ≥4 glasses or ≥40 grams/day for men, and ≥2 glasses or 20 grams/day for women.
Data regarding coronary heart disease (CHD), heart failure (HF), and diabetes mellitus (DM) were obtained through the records of general practitioners and hospitals.
Medication use was verified through the medical records of pharmacies. The use of cardiac therapy and lipid-reducing agents was categorized and defined according to the World Health Organization Anatomical Therapeutic Chemical (WHO ATC) classifications. Renal function was defined as estimated glomerular filtration rate (eGFR), calculated based on the creatinine and Cystatin C values [2].

References

1. Ikram MA, Brusselle G, Ghanbari M, Goedegebure A, Ikram MK, Kavousi M, Kieboom BCT, Klaver CCW, de Knegt RJ, Luik AI, Nijsten TEC, Peeters RP, van Rooij FJA, Stricker BH, Uitterlinden AG, Vernooij MW, Voortman T. Objectives, design and main findings until 2020 from the Rotterdam Study. Eur J Epidemiol. 2020;35(5):483-517. https://doi.org/10.1007/s10654-020-00640-5
2. Schlipak MG, Matsushita K, Ärnöv J, Inker LA, Katz R, Polkinghorne KR, Rothenbacher D, Sarnak MJ, Astor BC, Coresh J, Levey AS, Gansevoort RT, CKD Prognosis Consortium. Cystatin C versus Creatinine

in Determining Risk Based on Kidney Function. N Engl J Med. 2013 Sep 5;369(10):932-43.
https://doi.org/10.1056/NEJMoa1214234
