# Supplementary material for: Immunothrombosis and new-onset atrial fibrillation in the general population: the Rotterdam Study
Source: Clin Res Cardiol. 2021 Sep 24;111(1):96–104. doi: 10.1007/s00392-021-01938-4 (PMC8766396; doi:10.1007/s00392-021-01938-4)

Immunothrombosis and new-onset atrial fibrillation in the general population: the Rotterdam Study

Martijn J. Tilly[a], MD, Sven Geurts[a], MD, Samantha J. Donkel[b], MD, M. Arfan Ikram[a], Phd, Natasja M.S. de Groot[c], PhD, Moniek P.M. de Maat[b], PhD, *Maryam Kavousi[a], PhD

[a] Department of Epidemiology, Erasmus MC University Medical Center Rotterdam, Rotterdam, The Netherlands

[b] Department of Hematology, Erasmus MC University Medical Center Rotterdam, Rotterdam, The Netherlands

[c] Department of Cardiology, Erasmus MC University Medical Center Rotterdam, Rotterdam, The Netherlands

Corresponding author: Maryam Kavousi

Erasmus MC, University Medical Center Rotterdam, office Na-2714

PO Box 2040, 3000 CA Rotterdam, The Netherlands

Telephone Number: +31 10 7043997

Email: m.kavousi@erasmusmc.nl

Journal: Clinical Research in Cardiology

Online Resource 2. Flowchart for the inclusion of the study population

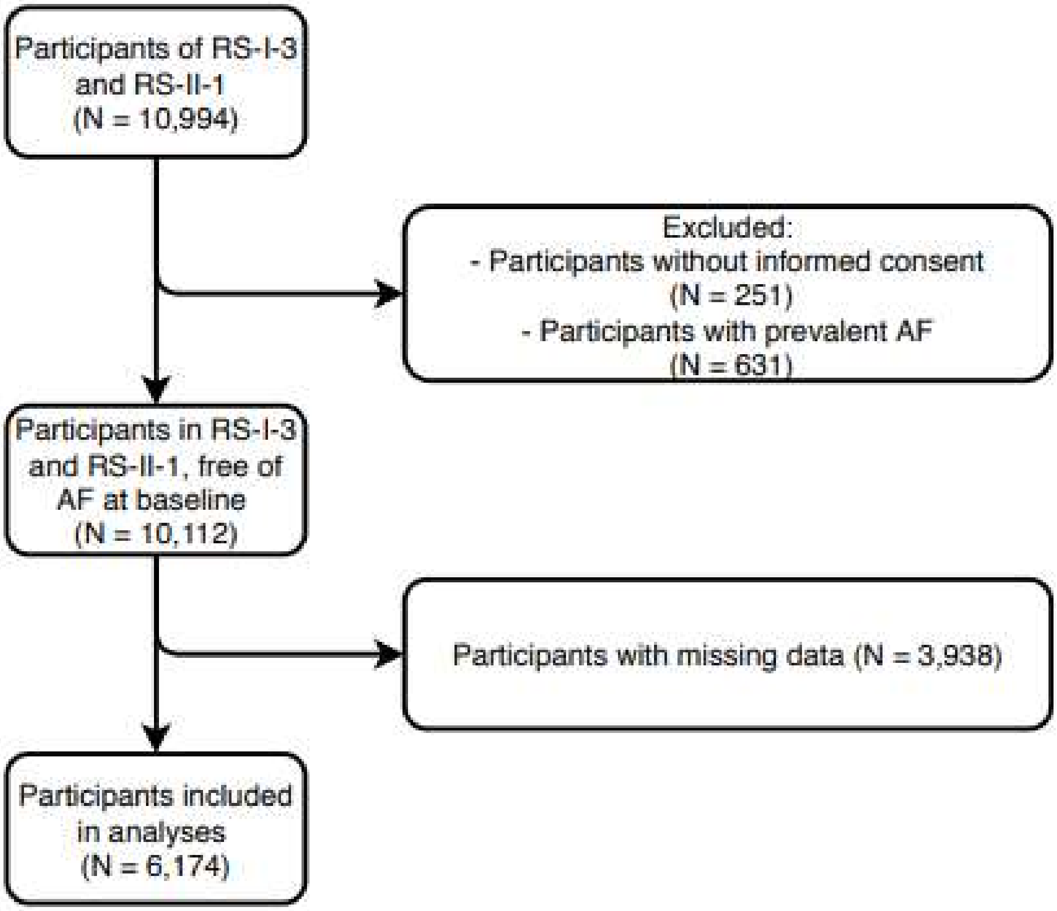

Supplement: Supplementary file 2 — Supplementary file2 (PDF 382 kb) [file 392_2021_1938_MOESM2_ESM.pdf]
