# Supplementary material for: Immunothrombosis and new-onset atrial fibrillation in the general population: the Rotterdam Study
Source: Clin Res Cardiol. 2021 Sep 24;111(1):96–104. doi: 10.1007/s00392-021-01938-4 (PMC8766396; doi:10.1007/s00392-021-01938-4)
Supplement: Supplementary file 3 — Supplementary file3 (PDF 396 kb) [file 392_2021_1938_MOESM3_ESM.pdf]

Immunothrombosis and new-onset atrial fibrillation in the general population: the Rotterdam Study

Martijn J. Tilly[a], MD, Sven Geurts[a], MD, Samantha J. Donkel[b], MD, M. Arfan Ikram[a], Phd, Natasja M.S. de Groot[c], PhD, Moniek P.M. de Maat[b], PhD, *Maryam Kavousi[a], PhD

[a] Department of Epidemiology, Erasmus MC University Medical Center Rotterdam, Rotterdam, The Netherlands

[b] Department of Hematology, Erasmus MC University Medical Center Rotterdam, Rotterdam, The Netherlands

[c] Department of Cardiology, Erasmus MC University Medical Center Rotterdam, Rotterdam, The Netherlands

Corresponding author: Maryam Kavousi

Erasmus MC, University Medical Center Rotterdam, office Na-2714

PO Box 2040, 3000 CA Rotterdam, The Netherlands

Telephone Number: +31 10 7043997

Email: m.kavousi@erasmusmc.nl

Journal: Clinical Research in Cardiology

Online Resource 3. Baseline characteristics of the study population, stratified by sex

	Men (N=2,654)	Women (N=3,520)	P-value
Age, years	68.4 ± 7.7	69.6 ± 8.4	P<0.001
BMI (kg/m^2)	26.5 ± 3.7	27.3 ± 4.4	p<0.001
Current smoker, N(%)	642 (24.2%)	605 (17.2%)	p<0.001
Prevalent DM, N(%)	396 (14.9%)	401 (11.4%)	p<0.001
Prevalent CHD, N(%)	377 (14.2%)	141 (4.0%)	p<0.001
Prevalent HF, N(%)	81 (3.1%)	82 (2.3%)	p=0.048
Prevalent hypertension, N(%)	1,811 (68.2%)	2,351 (66.8%)	p=0.12
eGFR (mL/min/1.73m^2)	76.2 ± 15.9	74.0 ± 15.5	p<0.001
Systolic blood pressure (mmHg)	143.9 ± 20.8	142.8 ± 21.4	p=0.035
Diastolic blood pressure (mmHg)	78.4 ± 11.5	75.6 ± 10.8	p<0.001
Blood pressure lowering medication, N(%)	893 (33.6%)	1,279 (36.3%)	p=0.014
Daily alcohol intake (grams)	11.4 (20.9)	1.4 (10.1)	p<0.001
Prevalent alcohol abuse, N(%)	286 (10.8%)	653 (18.6%)	p<0.001
Use of cardiac therapy, N(%)	226 (8.5%)	258 (7.3%)	p=0.047
Lipid-reducing agents, N(%)	366 (13.8%)	447 (12.7%)	p=0.13
Thrombocyte count (x10^9/L)	241.4 ± 55.7	269.4 ± 57.4	p<0.001
Leucocyte count (x10^9/L)	7.0 ± 1.9	6.7 ± 1.9	p<0.001
Lymphocyte count (x10^9/L)	2.6 ± 0.9	2.6 ± 1.0	p=0.528
Lymphocyte percentage of leucocytes (%)	37.5 ± 7.5	39.4 ± 8.0	p<0.001
Platelet/lymphocyte ratio	99.1 ± 32.3	111.6 ± 35.1	p<0.001
Total cholesterol (mmol/L)	5.5 ± 1.0	6.0 ± 1.0	p<0.001
HDL cholesterol (mmol/L)	1.2 ± 0.3	1.5 ± 0.4	p<0.001
CRP (mg/L)	1.7 (3.0)	1.8 (3.0)	p=0.040
Plasma fibrinogen (g/L)	3.7 (1.1)	3.9 (1.1)	p<0.001
Plasma VWF:Ag (IU/mL)	1.20 (0.65)	1.18 (0.66)	p=0.12
ADAMTS13 activity (%)	87.3 ± 16.9	94.8 ± 17.6	p<0.001
MPO-DNA complex (mAU/mL)	54 (47)	53 (43)	p=0.073

Categorical data presented as N (%).
Continuous data presented as mean ± SD for normally distributed data, or median (IQR) for skewed distributed data.
P-value refers to the difference in characteristics between men and women. Significance was tested using Independent Samples T-tests for normally distributed variables, Mann-Whitney U test for skewed distributed variables, or Pearson's Chi-Squared tests for categorical data.
BMI, Body Mass Index; DM, diabetes mellitus; CHD, coronary heart disease; HF, heart failure; eGFR, estimated glomerular filtration rate; HDL, high density lipoprotein; CRP, C-reactive protein; vWF:Ag, von Willebrand Factor antigen; ADAMTS13, A Disintegrin And Metalloprotease with ThromboSpondin motif repeats 13. Alcohol abuse is defined as ≥4 alcoholic consumptions/day for men, and ≥2 for women.
