# Supplementary material for: Immunothrombosis and new-onset atrial fibrillation in the general population: the Rotterdam Study
Source: Clin Res Cardiol. 2021 Sep 24;111(1):96–104. doi: 10.1007/s00392-021-01938-4 (PMC8766396; doi:10.1007/s00392-021-01938-4)
Supplement: Supplementary file 4 — Supplementary file4 (PDF 464 kb) [file 392_2021_1938_MOESM4_ESM.pdf]

Immunothrombosis and new-onset atrial fibrillation in the general population: the Rotterdam Study

Martijn J. Tilly[a], MD, Sven Geurts[a], MD, Samantha J. Donkel[b], MD, M. Arfan Ikram[a], Phd, Natasja M.S. de Groot[c], PhD, Moniek P.M. de Maat[b], PhD, *Maryam Kavousi[a], PhD

[a] Department of Epidemiology, Erasmus MC University Medical Center Rotterdam, Rotterdam, The Netherlands

[b] Department of Hematology, Erasmus MC University Medical Center Rotterdam, Rotterdam, The Netherlands

[c] Department of Cardiology, Erasmus MC University Medical Center Rotterdam, Rotterdam, The Netherlands

Corresponding author: Maryam Kavousi

Erasmus MC, University Medical Center Rotterdam, office Na-2714

PO Box 2040, 3000 CA Rotterdam, The Netherlands

Telephone Number: +31 10 7043997

Email: m.kavousi@erasmusmc.nl

Journal: Clinical Research in Cardiology

Online Resource 4. Association between markers of immunothrombosis and new-onset atrial fibrillation, stratified by sex

	Men		Women	
	Model 1 HR (95% CI)	Model 2 HR (95% CI)	Model 1 HR (95% CI)	Model 2 HR (95% CI)
Fibrinogen (g/L)	1.02 (0.92 – 1.13)	0.98 (0.88 – 1.09)	1.03 (0.92 – 1.16)	1.04 (0.93– 1.17)
vWF:Ag (IU/mL)	1.04 (0.93 – 1.16)	1.01 (0.91 – 1.13)	1.06 (0.95 – 1.18)	1.05 (0.94 – 1.17)
ADAMTS13 (%)	1.00 (0.99 – 1.00)	1.00 (0.99 – 1.00)	1.01 (1.00 – 1.01)	1.01 (1.00 – 1.01)
vWF:Ag/ADAMTS13 ratio	1.06 (0.96– 1.17)	1.04 (0.94 – 1.15)	0.97 (0.86 – 1.09)	0.96 (0.85 – 1.08)
MPO-DNA complex (mAU/mL)	0.98 (0.88 – 1.09)	0.98 (0.88 – 1.09)	1.04 (0.94 – 1.15)	1.04 (0.94 – 1.15)

Presented estimated are hazard ratio (95% confidence interval) per one standard deviation increase in each marker.
Model 1 is adjusted for age, and Rotterdam Study cohort. Model 2 is additionally adjusted for current smoking, hypertension, alcohol use, renal function, use of cardiac therapy, use of lipid-reducing agents, prevalent diabetes mellitus, prevalent heart failure, and prevalent coronary heart disease.
HR, Hazard Ratio; CI, Confidence Interval; vWF:Ag, von Willebrand Factor antigen; ADAMTS13, A Disintegrin And Metalloprotease with ThromboSpondin motif repeats 13.
