# Supplementary material for: Immunothrombosis and new-onset atrial fibrillation in the general population: the Rotterdam Study
Source: Clin Res Cardiol. 2021 Sep 24;111(1):96–104. doi: 10.1007/s00392-021-01938-4 (PMC8766396; doi:10.1007/s00392-021-01938-4)
Supplement: Supplementary file 5 — Supplementary file5 (PDF 705 kb) [file 392_2021_1938_MOESM5_ESM.pdf]

Immunothrombosis and new-onset atrial fibrillation in the general population: the Rotterdam Study

Martijn J. Tilly[a], MD, Sven Geurts[a], MD, Samantha J. Donkel[b], MD, M. Arfan Ikram[a], Phd, Natasja M.S. de Groot[c], PhD, Moniek P.M. de Maat[b], PhD, *Maryam Kavousi[a], PhD

[a] Department of Epidemiology, Erasmus MC University Medical Center Rotterdam, Rotterdam, The Netherlands

[b] Department of Hematology, Erasmus MC University Medical Center Rotterdam, Rotterdam, The Netherlands

[c] Department of Cardiology, Erasmus MC University Medical Center Rotterdam, Rotterdam, The Netherlands

Corresponding author: Maryam Kavousi

Erasmus MC, University Medical Center Rotterdam, office Na-2714

PO Box 2040, 3000 CA Rotterdam, The Netherlands

Telephone Number: +31 10 7043997

Email: m.kavousi@erasmusmc.nl

Journal: Clinical Research in Cardiology

Online Resource 5. Association between markers of immunothrombosis and incident atrial fibrillation stratified by sex, per quartile

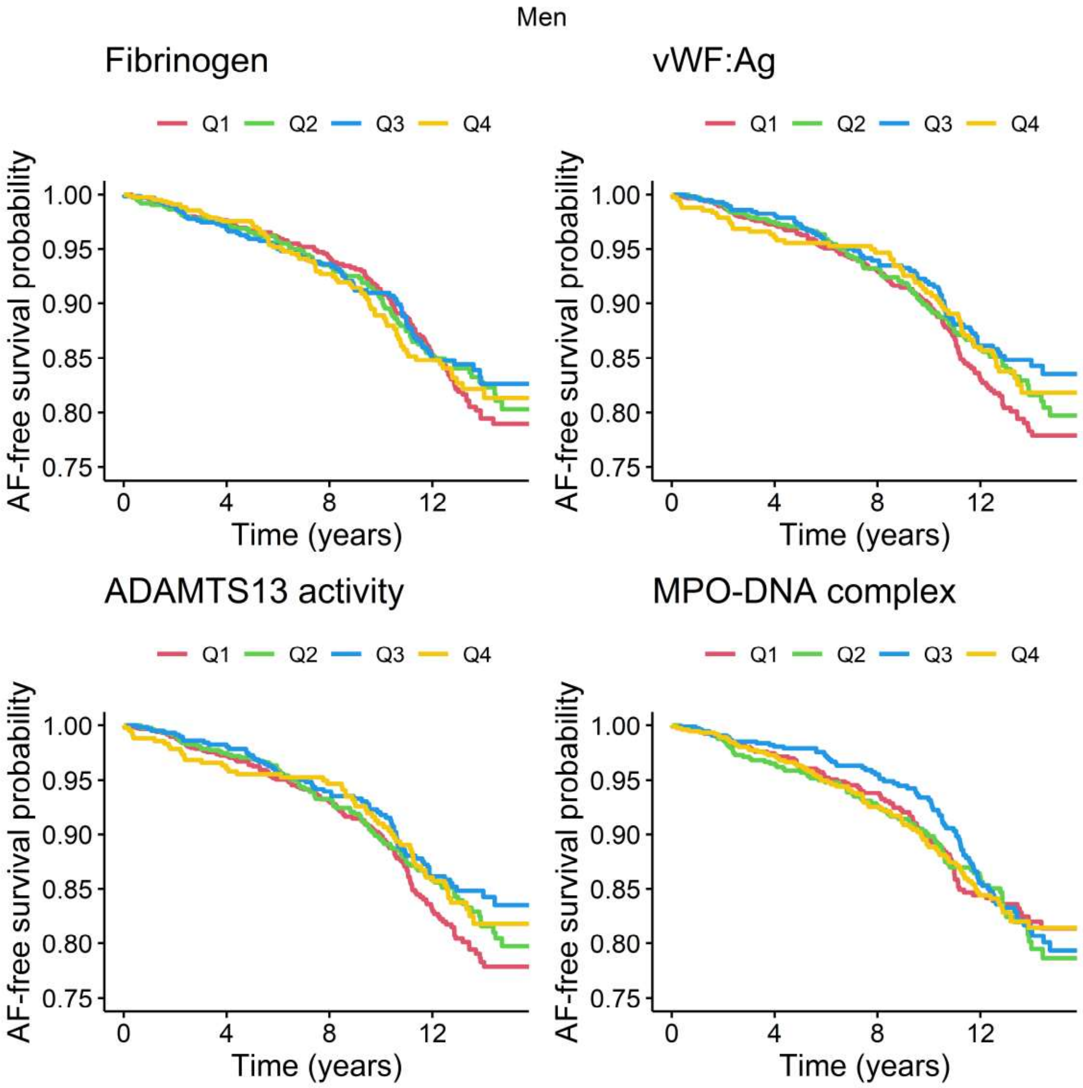

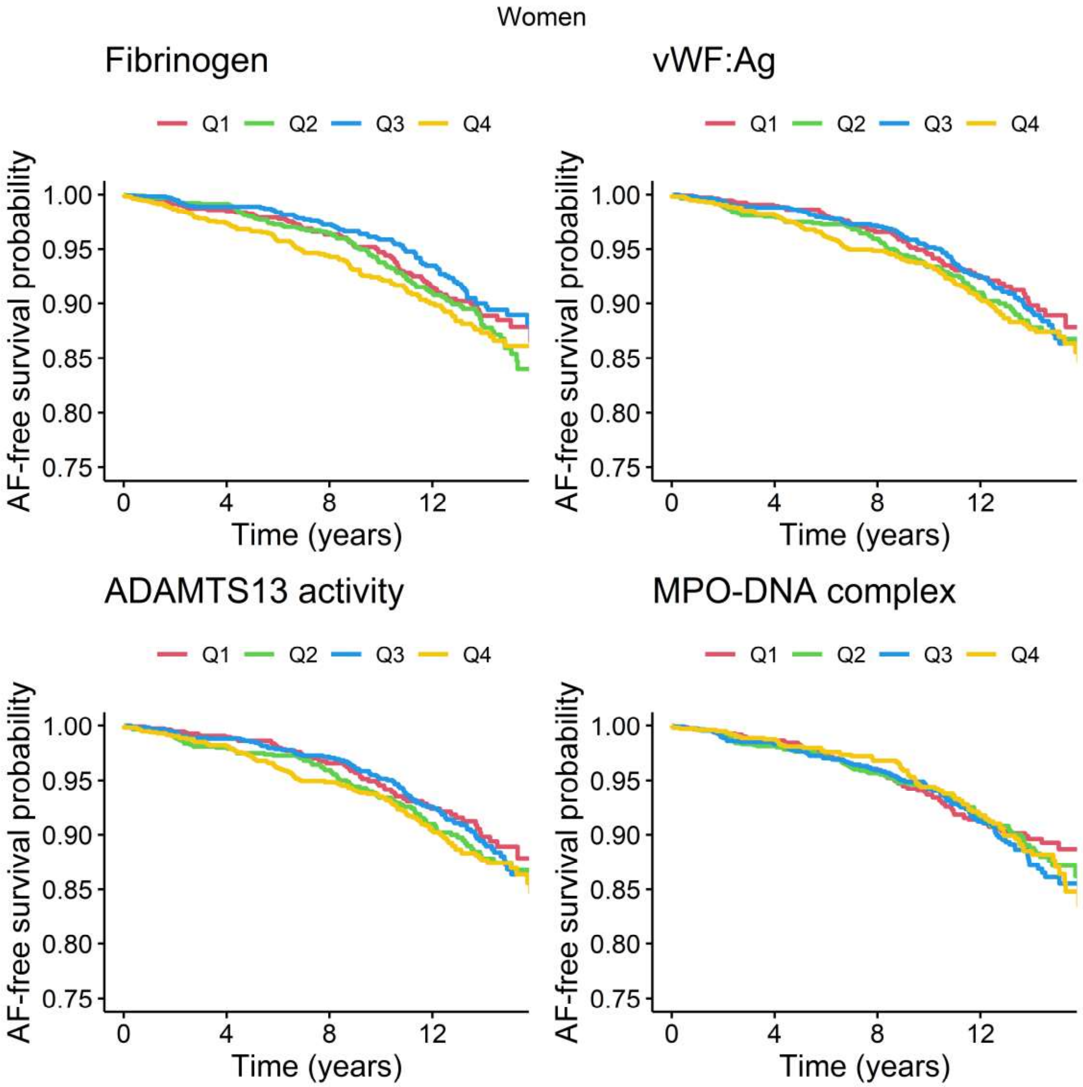

Adjusted for age, Rotterdam Study cohort, current smoking, alcohol use, renal function, hypertension, use of cardiac therapy, use of lipid-reducing agents, prevalent diabetes mellitus, prevalent heart failure, and prevalent coronary heart disease. Quartiles fibrinogen: ≤ 3.30 g/L, 3.31-3.80 g/L, 3.81-4.40 g/L, and ≥4.41 g/L. vWF:Ag: ≤0.93 IU/mL, 0.94-1.20 IU/mL, 1.21-1.60 IU/mL, and ≥1.61 IU/mL.. ADAMTS13: ≤80.31%, 80.32-91.00%, 91.01-101.75%, and ≥101.76%. MPO-DNA complex: ≤42 mAU/mL,42-53 mAU/mL, 54-87 mAU/mL, and 88 mAU/mL.
